# Clinical Presentations, Treatments, and Complications of Ileal Perforation at a Tertiary Center: A Cross-Sectional Study

**DOI:** 10.7759/cureus.72027

**Published:** 2024-10-21

**Authors:** Eesha Ashok, Nishith M Ekka, Dipendra K Sinha, Kumar Gaurav, Binay Kumar, Neyaz Ahmad, Deepak Kumar, Sumit Chandra, Prem P Choudhary

**Affiliations:** 1 Surgery, Srirama Chandra Bhanja (SCB) Medical College and Hospital, Cuttack, IND; 2 General Surgery, Rajendra Institute of Medical Sciences, Ranchi, IND

**Keywords:** enteric perforation, ileal perforation, ileostomy, pyoperitoneum, resection and anastomosis, stoma related complications, widal test

## Abstract

Background

Ileal perforations represent one of the most common surgical emergencies in India, associated with significant morbidity and mortality rates. The causes of these perforations include infections such as tuberculosis and enteric fever, as well as malignancy and trauma. Management options encompass ileostomy, resection with anastomosis, and primary closure.

Objective

The objective of this study is to evaluate the clinical profile, etiology, management strategies, and complications associated with ileal perforation.

Methodology

A total of 70 patients with ileal perforation were included in the study. The proportions of various etiological factors, surgical interventions, and their associated complications were analyzed. The study aimed to determine whether ileostomy leads to fewer complications compared to primary repair.

Results

Ileal perforation is more prevalent in males than in females, with an incidence ratio of 2.68:1, predominantly affecting individuals in their 30s. Antituberculosis treatment does not consistently protect against perforation. Delays in presentation are linked to a higher likelihood of developing pyoperitoneum, increased chances of stoma creation (with a mean delay of 2.50 days in the closure group compared to 4.98 days in the ileostomy group), and greater mortality. Closure was performed in 18 patients (25.7%), while 52 patients (74.3%) underwent stoma creation. The duration of surgery was longer in the closure group (2.889 hours) than in the ileostomy group (2.635 hours) and also greater in the mortality group (three hours) compared to the non-mortality group (2.64 hours). The mean number of perforations was 1.59 ± 0.970, with 1.33 ± 0.840 in the closure group and 1.67 ± 1.004 in the ileostomy group. A higher number of perforations is associated with an increased likelihood of stoma creation. The most common cause of perforation was nonspecific, accounting for 40% of cases. The most frequently encountered complication was wound infection, occurring in 42.9% of patients. The mean duration of hospital stay was shorter in the ileostomy group (9.50 ± 5.500 days) than in the closure group (17.22 ± 19.219 days). Death occurred in 18.6% of cases, with higher rates observed in males (8:5), patients with delayed presentation, and elderly patients. A significant p-value was found in relation to leaks and fecal fistulas.

Conclusions

No significant difference was observed in complications associated with the various surgical procedures performed. However, a delay in presentation is linked to a higher rate of complications and increased mortality.

## Introduction

Perforation refers to a full-thickness breach of the bowel wall, allowing intestinal contents to leak into the peritoneal cavity. Ileal perforation peritonitis is the fifth most common cause of abdominal emergencies and significantly contributes to morbidity and mortality in surgical practice [1]. Necrosis of Peyer’s patches is a key factor leading to intestinal perforation and subsequent bleeding [2]. Enteric fever is the primary cause of ileal perforation, accounting for 20-70% of cases reported in various studies [1,3-6]. Tuberculosis has been associated with 3-79.6% of ileal perforation cases [4,7], and in developing countries, typhoid and tuberculosis remain the leading causes [5]. Other causes include radiation enteritis, foreign bodies, and trauma [1,3,4].

Although tuberculosis can affect any part of the intestine, it predominantly targets the ileum and proximal colon due to factors such as stasis in the area, a high absorption rate, abundant lymphoid tissue, and the more complete digestion that facilitates prolonged contact between the organism and the mucosal lining [8,9]. Over time, the management of perforations has shifted from conservative to surgical approaches, as conservative treatment is associated with higher mortality rates [5,10]. Surgical options for treating ileal perforations include primary repair, resection and anastomosis, perforation repair with ileo-transverse anastomosis, ileostomy, and single-layer repair with an omental patch. The types of stoma utilized include loop ileostomy, end ileostomy, and double-barrel ileostomy.

Stoma-related complications may include prolapse, retraction, ischemia, stenosis, parastomal hernia, bleeding, and fistulation [11]. Wound infection is the most common complication following surgery for ileal perforations, with incidence rates ranging from 10.7% to 71.4% [12,13]. Other complications encompass wound dehiscence, burst abdomen, residual abscess, fecal fistula, reperforation, septicemia, paralytic ileus, respiratory issues, bedsores, peritonitis, incisional hernia, electrolyte imbalance, and mortality [1,5-7,12-18]. The risk of fistula formation increases when sutures are placed in an aseptic environment. Additionally, stoma management poses challenges in developing countries, where many patients experience peristomal skin excoriation [12]. Therefore, this study aims to investigate the outcomes and complications of ileostomy closure, addressing the lack of available data in our region.

## Materials and methods

This analytical cross-sectional study was conducted in the Department of General Surgery at Rajendra Institute of Medical Sciences (RIMS), Ranchi, India, from February 2023 to May 2024, following approval from the Institutional Ethics Committee of RIMS, Ranchi (memo 48 dated 2/16/2023). All patients diagnosed with ileal perforation were included in the study.

Patient population

All patients who presented to RIMS, Ranchi, with a diagnosis of intraoperative ileal perforation and no associated organ damage were included in the study.

Sample size

The prevalence of ileal perforation varies between 0.8% and 39%. Consequently, we adopted an average prevalence of 20% [19]. Using the formula n = z2*p*(1-p)/d2, with P = 20, D = 10, and a study power of 90, the calculated sample size was 61.4. Accounting for a 10% loss to follow-up, we included a total of 70 patients in our study.

Inclusion criteria

All patients diagnosed with ileal perforation, aged between 10 and 80 years, who were treated during the study period were included in the study.

Exclusion criteria

Patients were excluded if they had any concurrent clinical conditions alongside ileal perforation, uncontrolled diabetes mellitus, were not expected to survive more than 24 hours post-surgery, lacked available histopathological examination (HPE) or Widal reports, or if they were lost to follow-up.

Data collection method

All consecutive patients diagnosed with ileal perforation in the Department of General Surgery were enrolled in this study. Upon confirmation of ileal perforation during laparotomy, the investigators were notified. Data on age, surgery duration, number of complications, length of hospital stay, duration of abdominal distension, and duration of abdominal pain were recorded as continuous variables and expressed as means with SDs. Data on sex, incidence of diarrhea, absolute constipation, fever, vomiting, presence of pyoperitoneum, significant medical history, surgical complications, resection of the segment, ileostomy, and closure were recorded as categorical variables and expressed as frequencies and percentages. The types of ileostomy, histopathological findings, and previous surgical procedures were documented as nominal variables and presented as frequencies and percentages. To minimize bias, data from all six units of the department was collected. The duration of surgery was calculated from the incision to closure.

Statistical analysis

Patients who underwent primary repair or ileostomy were divided into two groups: Groups A and B. The mean and SD were calculated for age, surgery duration, number of complications, length of hospital stay, duration of abdominal distension, and duration of abdominal pain. Frequencies and percentages were determined for sex, incidence of diarrhea, absolute constipation, fever, vomiting, presence of pyoperitoneum, significant medical history, surgical complications, resection of the segment, ileostomy, and closure. The types of ileostomy, histopathological findings, and previous surgical procedures were also recorded. A 2 × 2 contingency table was constructed, with the groups serving as the exposure variable and complications as the outcomes. The chi-square test was employed to compare differences between the two groups. Data were entered into Microsoft Excel (Microsoft Corporation, Redmond, WA, USA) and subsequently exported to IBM SPSS Statistics for Windows, Version 27.0 (Released 2020; IBM Corp., Armonk, NY, USA) for statistical analysis.

## Results

A total of 70 patients with intraoperatively confirmed ileal perforation were included in the study after providing informed consent. All patients received preoperative resuscitation, a single intravenous dose of 1 g cefoperazone, and 100 ml of metronidazole. Nasogastric tube decompression and urine output monitoring via urethral catheterization were performed on all patients. The mean age of the patients was 40.83 years (SD: 18.338). Among the 70 patients, 51 (72.9%) were male and 19 (27.1%) were female, resulting in a male-to-female ratio of 2.68. Table 1 illustrates the sex ratio across different age groups.

**Table 1:** Age range and sex distribution

Age range (years)	Cases	Percentage	Male	Percentage	Female	Percentage
10-20	10	14.28%	10	14.28%	0	0%
21-30	19	27.14%	14	20%	5	7.10%
31-40	8	11.42%	4	5.70%	4	5.70%
41-50	8	11.42%	4	5.70%	4	5.70%
51-60	14	20%	10	14.28%	4	5.70%
61-70	7	10%	7	10%	0	0%
>70	4	5.70%	2	2.80%	2	2.80%

Abdominal distension was noted in all 70 patients (100%), with an average duration of 3.23 ± 2.509 days, ranging from one to 15 days. Additionally, all patients experienced abdominal pain (100%), with an average duration of 4.76 ± 4.493 days, lasting between one and 25 days. In those with tuberculosis, the average duration of abdominal pain was 4.5 days (range: one to 15 days), while for typhoid perforation, it was 4.44 days (range: one to 16 days). Table 2 presents the average pain durations for various etiologies.

**Table 2:** Mean duration according to etiology

Etiology	Mean duration of pain in the abdomen (days)	SD	Range
Typhoid	4.44	4.082	1-16
Tuberculosis	4.5	3.942	1-15
Crohn’s	5	0	
Diverticulosis	6.5	4.95	3-10
Meckel’s	4	0	
Non-Hodgkin’s lymphoma	25	0	
Nonspecific	4.57	2.768	2-10
Trauma	1.4	0.816	1-3
Band adhesion	3	2.828	1-5
Tubulovillous adenoma	20	0	

**Figure 1:**
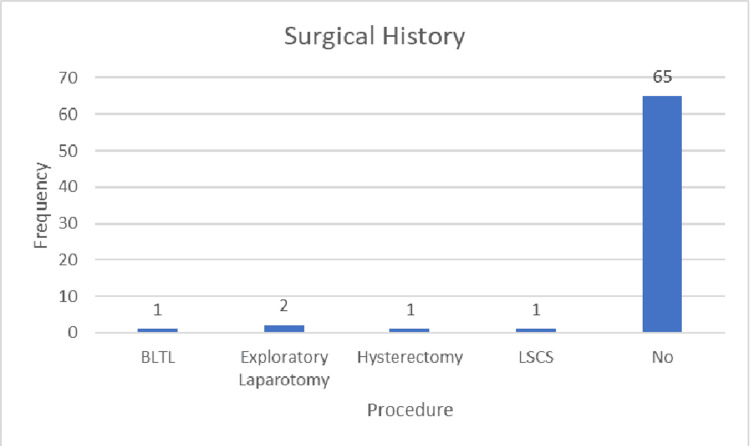
Surgical history

A history of fever was reported in 34 patients (48.6%), while vomiting occurred in 31 patients (44.3%), and diarrhea was noted in seven patients (10%). Absolute constipation, defined as the inability to pass stool or flatus, was present in 46 patients (65.7%). Among the 70 patients, two (2.9%) had type 2 diabetes mellitus and were on oral hypoglycemic agents, with one mortality recorded (50% of diabetic cases). Additionally, two patients (2.9%) had a history of tuberculosis and had received antituberculosis treatment (ATT). Furthermore, two patients (2.9%) had a history of hypertension and were on antihypertensive medication; no deaths occurred among these patients. Five patients had a history of previous surgeries: one underwent bilateral tubal ligation (BLTL), two had exploratory laparotomies (documentation for these cases was unavailable), one had a hysterectomy, and one had a lower segment cesarean section (LSCS). Figure 1 illustrates the surgical histories of the patients.

**Figure 2:**
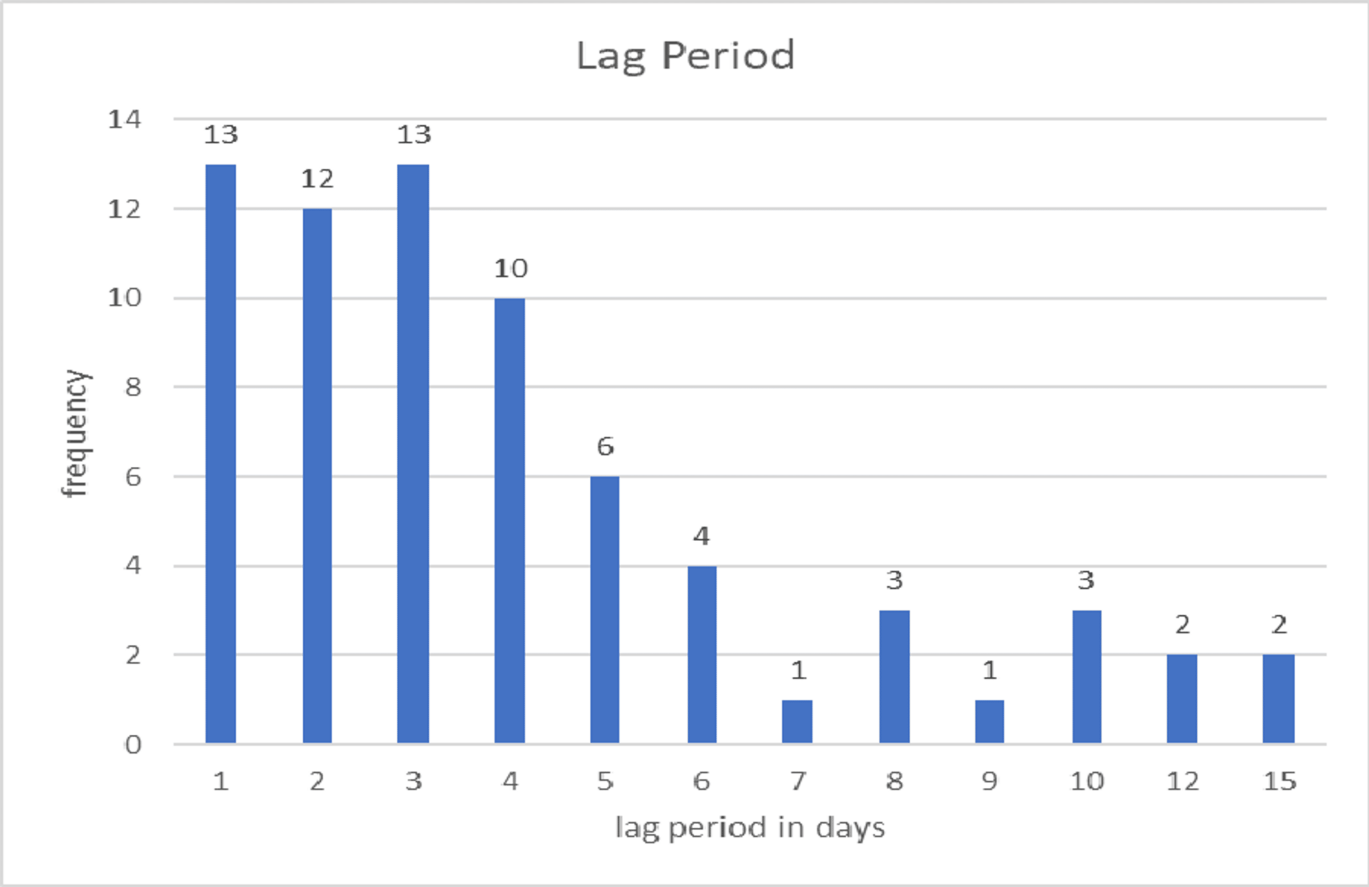
Frequency of lag period in days

Of the 70 patients, 25 presented within 48 hours, while the remaining patients presented after this time frame. The average delay before presentation was 4.02 days in the non-mortality group compared to 5.76 days in the mortality group. Among patients with pyoperitoneum, the delay averaged 5.75 days, whereas it was 3.22 days for those without pyoperitoneum. In the closure group, the average delay was 2.50 days, while in the ileostomy group, it was 4.98 days. Figure 2 illustrates the delay periods in days.

The duration of surgery was recorded in hours, with an average surgical time of 2.707 ± 0.6941 hours. For patients who underwent closure, the average duration was three hours, while those in the ileostomy group had an average time of 2.635 hours. In patients with pyoperitoneum, the average surgery time was 3.3 hours compared to 2.577 hours for those without pyoperitoneum. Among the 12 patients with pyoperitoneum, only one underwent primary closure, while the others had stomas created. The average surgery time was three hours in the mortality group, compared to 2.64 hours in the non-mortality group. Table 3 illustrates the relationship between surgery duration and the presence of pyoperitoneum.

**Table 3:** Duration of surgery and the presence of pyoperitoneum

Hours	Frequency	Percentage	Pyoperitoneum presence
1.5	1	1.40%	0
2	28	40.00%	1
3	32	45.70%	6
4	9	12.90%	5

**Figure 3:**
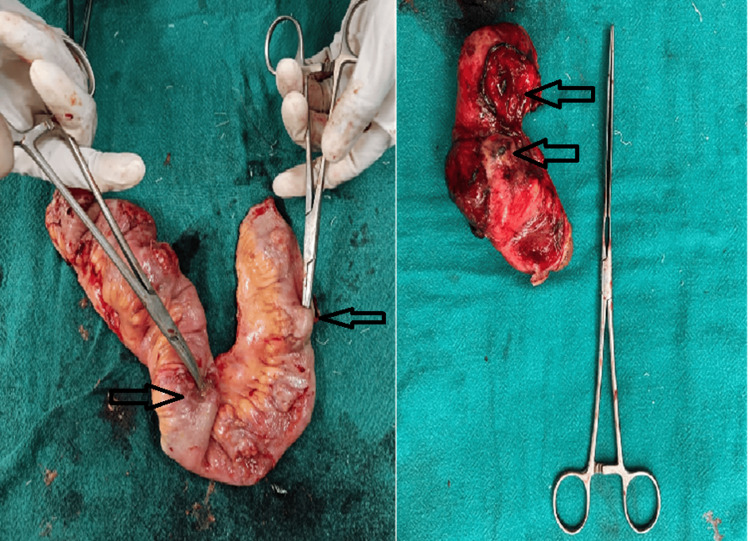
Multiple perforations The arrows indicate the locations of the perforations.

The mean number of perforations was 1.59 ± 0.970, with a maximum of five perforations observed. The distribution of perforations ranged from 1 to 5. In patients with pyoperitoneum, the mean number of perforations increased to 1.91. Specifically, 45 patients (64.3%) had a single perforation, 15 patients (21.4%) had two perforations, six patients (8.6%) had three perforations, two patients (2.9%) had four perforations, and two patients (2.9%) had five perforations. The mean number of perforations in the closure group was 1.33 ± 0.840, while it was 1.67 ± 1.00 in the ileostomy group. Table 4 presents the distance of perforations from the ileocecal junction, and Figure 3 illustrates cases with multiple perforations.

**Table 4:** Distance of perforation from ileocecal junction Values of “10, 12” indicate two perforations located at distances of 10 cm and 12 cm from the ileocecal junction, while “10.5” indicates a single perforation at a distance of 10.5 cm from the ileocecal junction.

Location from the ileocecal junction (cm)	Frequency	Percentage
10	3	4.30%
10, 10	1	1.40%
10, 12	1	1.40%
10, 20	1	1.40%
10, 25	1	1.40%
10.5	1	1.40%
15	7	10.00%
15, 20	1	1.40%
15.5	2	2.90%
16	1	1.40%
17	1	1.40%
20	7	10.00%
20, 21, 22, 30, 35	1	1.40%
20, 25	1	1.40%
20, 30	1	1.40%
20, 30, 30, 40, 41	1	1.40%
20, 50	1	1.40%
20.5	3	4.30%
25	5	7.10%
25, 30	1	1.40%
25, 35	1	1.40%
25.5	1	1.40%
30	6	8.60%
30, 70	1	1.40%
30.5	1	1.40%
35	3	4.30%
35, 70	1	1.40%
40	1	1.40%
5, 15	1	1.40%
5, 25	1	1.40%
5, 60	1	1.40%
50	3	4.30%
60	1	1.40%
7, 10, 12, 15	1	1.40%
7, 20	1	1.40%
70	2	2.90%
75, 105	1	1.40%
80	1	1.40%
Not assessed	1	1.40%
Total	70	100.00%

The farthest perforation from the ileocecal junction was located at 105 cm, while the closest was at 10 cm. In the closure group, the minimum distance of a perforation from the ileocecal junction was 15 cm; perforations closer than this were not primarily closed but were resected, with the distal segment closed and an end ileostomy created. The closest perforation overall was observed at 10 cm from the ileocecal junction. Perforations nearer than this were associated with cecal abnormalities and were excluded from the study. In one case of intestinal tuberculosis, the exact location of the perforation could not be determined due to intestinal clumping. Minimal adhesiolysis was performed, and the perforation was treated with a stoma, making it impossible to measure its distance from the ileocecal junction. Based on the caliber of the intestine, the presence of Peyer’s patches, and the reduced amount of stoma output, this section was assumed to be the terminal ileum and was included in this study.

**Figure 4:**
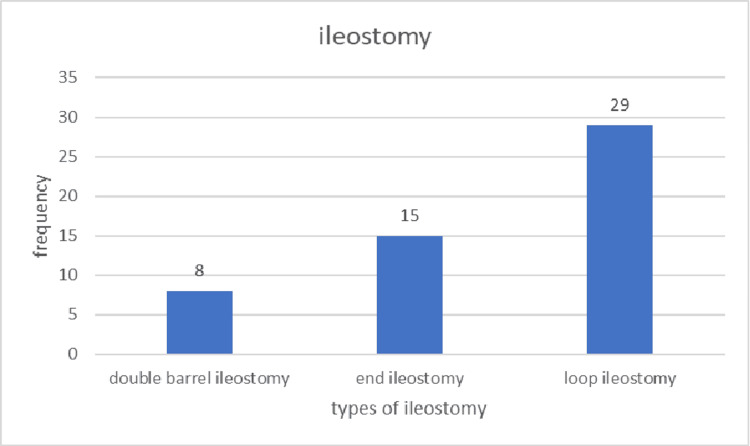
Types of ileostomy

**Figure 5:**
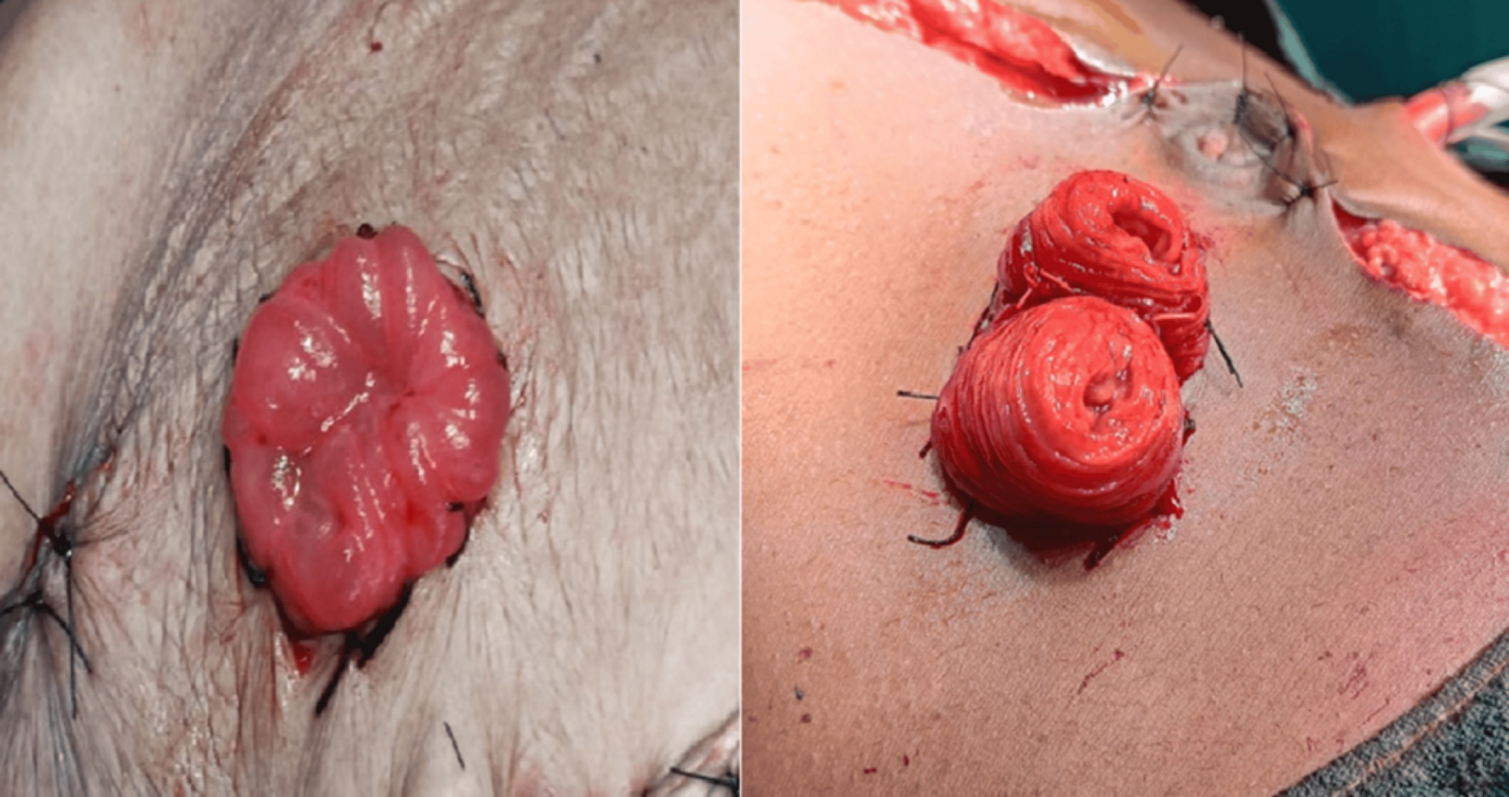
Types of ileostomy: loop ileostomy (left) and double-barrel ileostomy (right)

Segment resection was performed in 35 cases (50%). Among the 12 patients with pyoperitoneum, segment resection was performed in nine. For patients with a single perforation and pyoperitoneum, segment resection was conducted in three out of five patients, representing 60%. In patients with two or three perforations and pyoperitoneum, segment resection was performed in all cases. Of the 16 confirmed cases of typhoid disease, segment resection was performed in six patients, four of whom had two perforations. Among the 18 patients who underwent closure, nine underwent primary repair of the perforation, while the other nine underwent resection and anastomosis. Of the 52 patients who had ileostomy, eight (15.4%) underwent double-barrel ileostomy, 15 (28.8%) had end ileostomy, and 29 (55.8%) underwent loop ileostomy. Figure 4 illustrates the frequency of different types of ileostomy, while Figure 5 displays the various ileostomy types.

HPE revealed significant findings in 34 patients (48.6%), while 36 patients (51.4%) exhibited normal or non-significant results. Given that typhoid is endemic in India, antibody titers are often positive in the population. To minimize false-positive results, a Widal titer of dilution ≥ 1:320 was considered significant. Among the 16 cases that tested positive for Widal, HPE suggested typhoid in two cases.

The most common cause of ileal perforation identified in this study was nonspecific, accounting for 28 cases (40%). No instances of foreign body ingestion were reported. Typhoid was responsible for 16 cases (22.1%), making it the second most common cause of ileal perforation. Tuberculosis accounted for 12 cases (17.1%), ranking as the third most common cause. Cancers were identified in two cases (2.8%), including one case of tubulovillous adenoma and one of non-Hodgkin’s lymphoma, which caused perforation due to obstruction. Trauma was the cause in six cases (8.6%). Band adhesions accounted for two cases, while Crohn’s disease and Meckel’s diverticulum each accounted for one case (1.4%). Among the 12 diagnosed cases of tuberculosis, 11 exhibited positive histopathological findings. However, one patient, with a history of tuberculosis who had received ATT, did not show positive findings despite similar abdominal symptoms. Although the HPE was negative, this patient was diagnosed with tuberculosis based on clinical findings and medical history.

**Figure 6:**
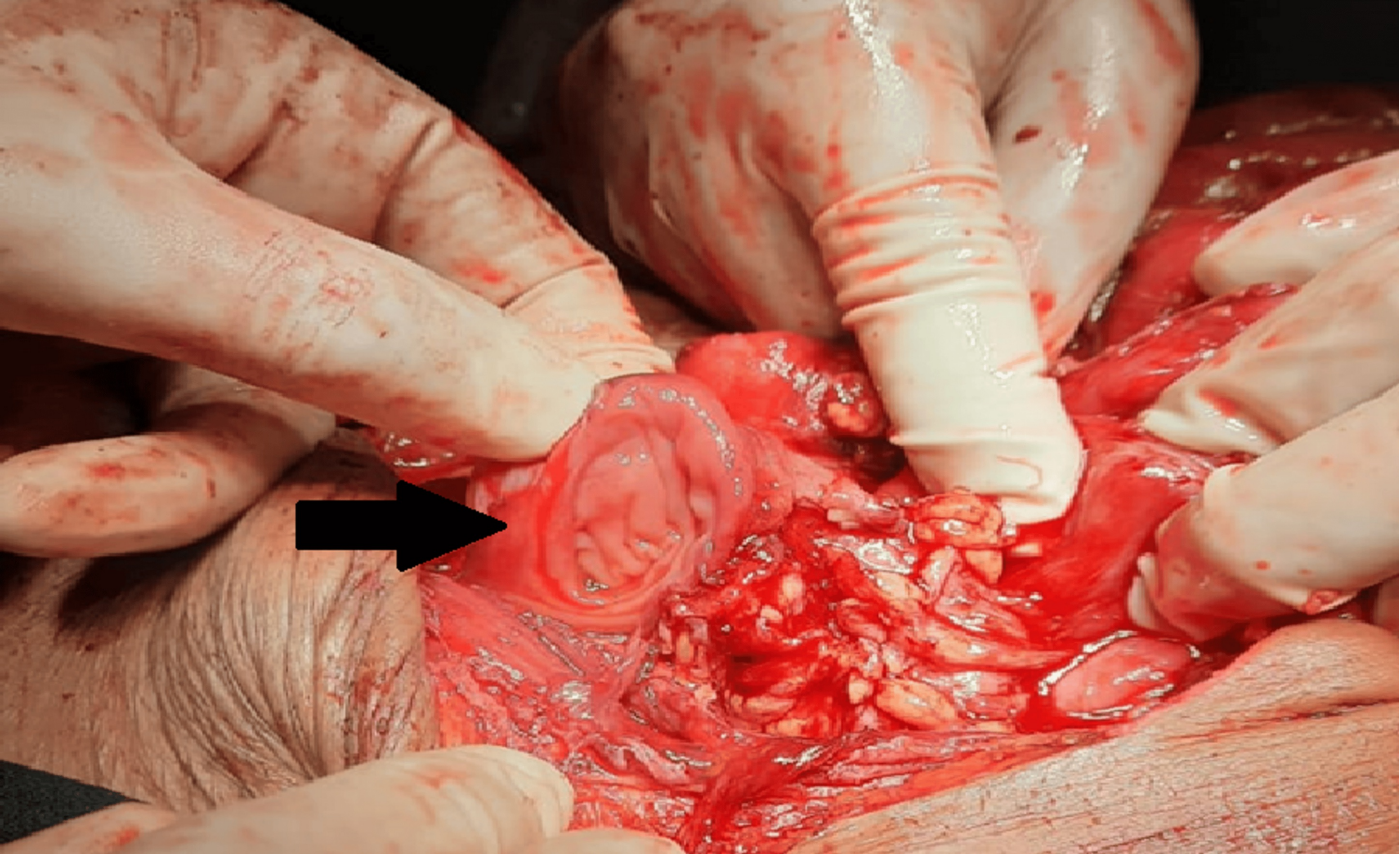
Traumatic ileal perforation (arrow)

**Figure 7:**
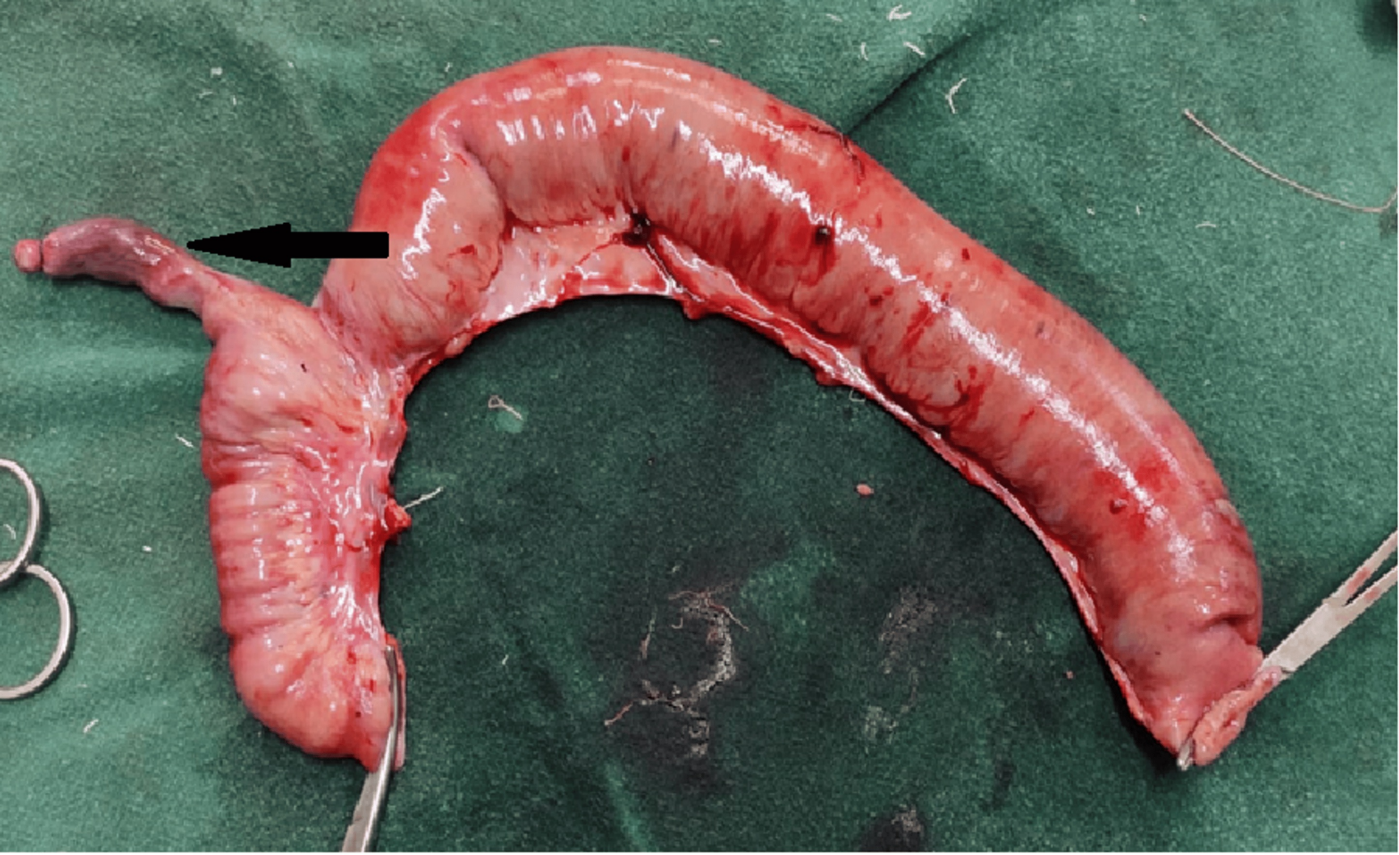
Meckel's diverticulum (arrow) with perforation at the base

**Figure 8:**
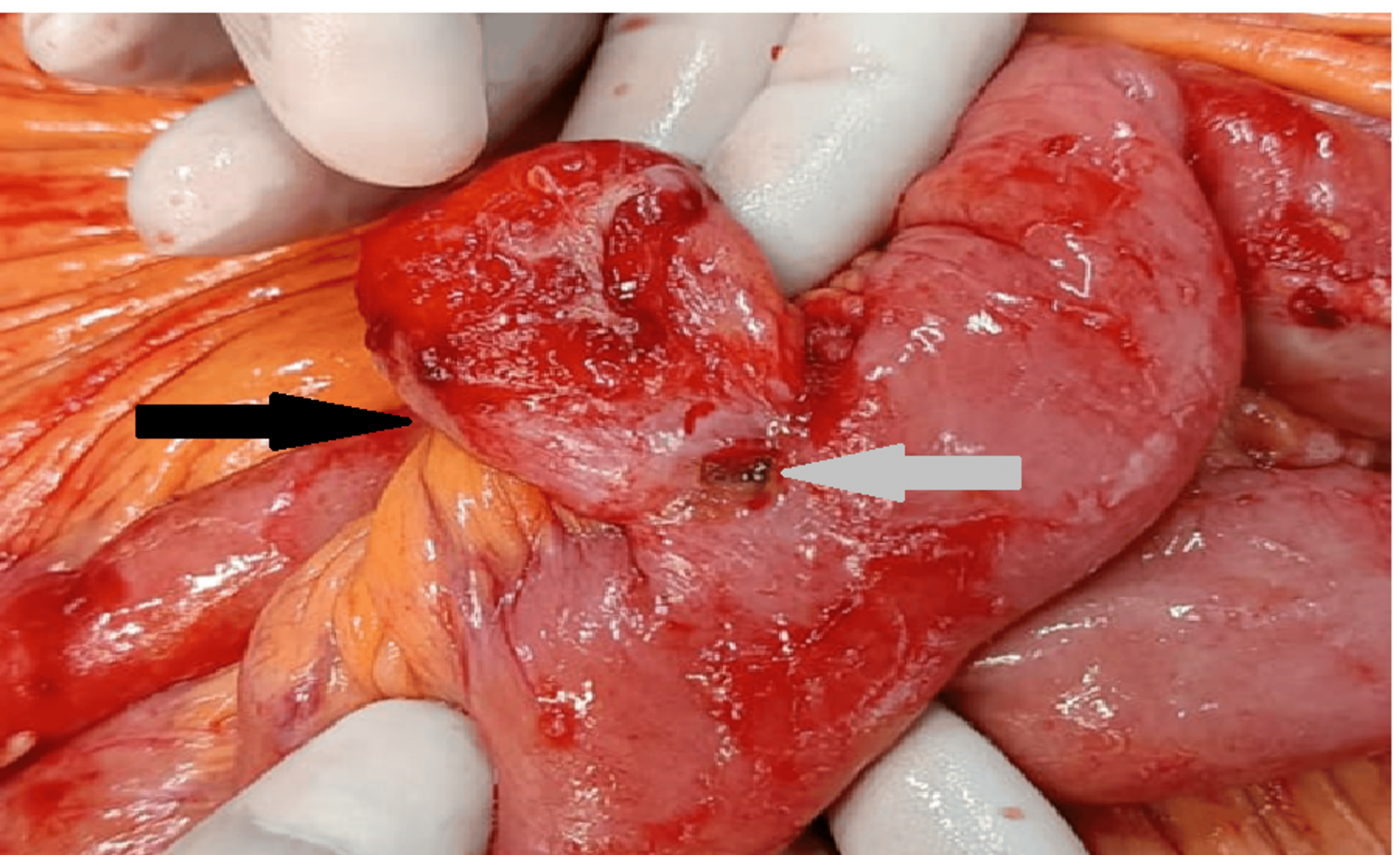
Diverticulum (black arrow) with perforation (white arrow) at the base

Table 5 presents the etiology-wise percentage of ileal perforations along with the corresponding sex ratios. Figure 6 depicts traumatic ileal perforation, Figure 7 illustrates perforation at the base of Meckel’s diverticulum, and Figure 8 shows perforation at the diverticulum’s base.

**Table 5:** Etiology of perforation

Etiology	Frequency	Percentage	Male	Female	Ratio (male: female)
Tuberculosis	12	17.10%	10	2	5:01
Band adhesion	2	2.90%	1	1	1:01
Crohn’s	1	1.40%	1		
Diverticulosis	2	2.90%	1	1	1:01
Typhoid	16	22.90%	13	3	4.33:1
Trauma	6	8.60%	5	1	5:01
Meckel’s diverticulum	1	1.40%	1	0	
Non-Hodgkin’s lymphoma	1	1.40%	1	0	
Non specific	28	40%	18	10	1.8:1
Tubulovillous adenoma	1	1.40%	1	0	

Complications

**Figure 9:**
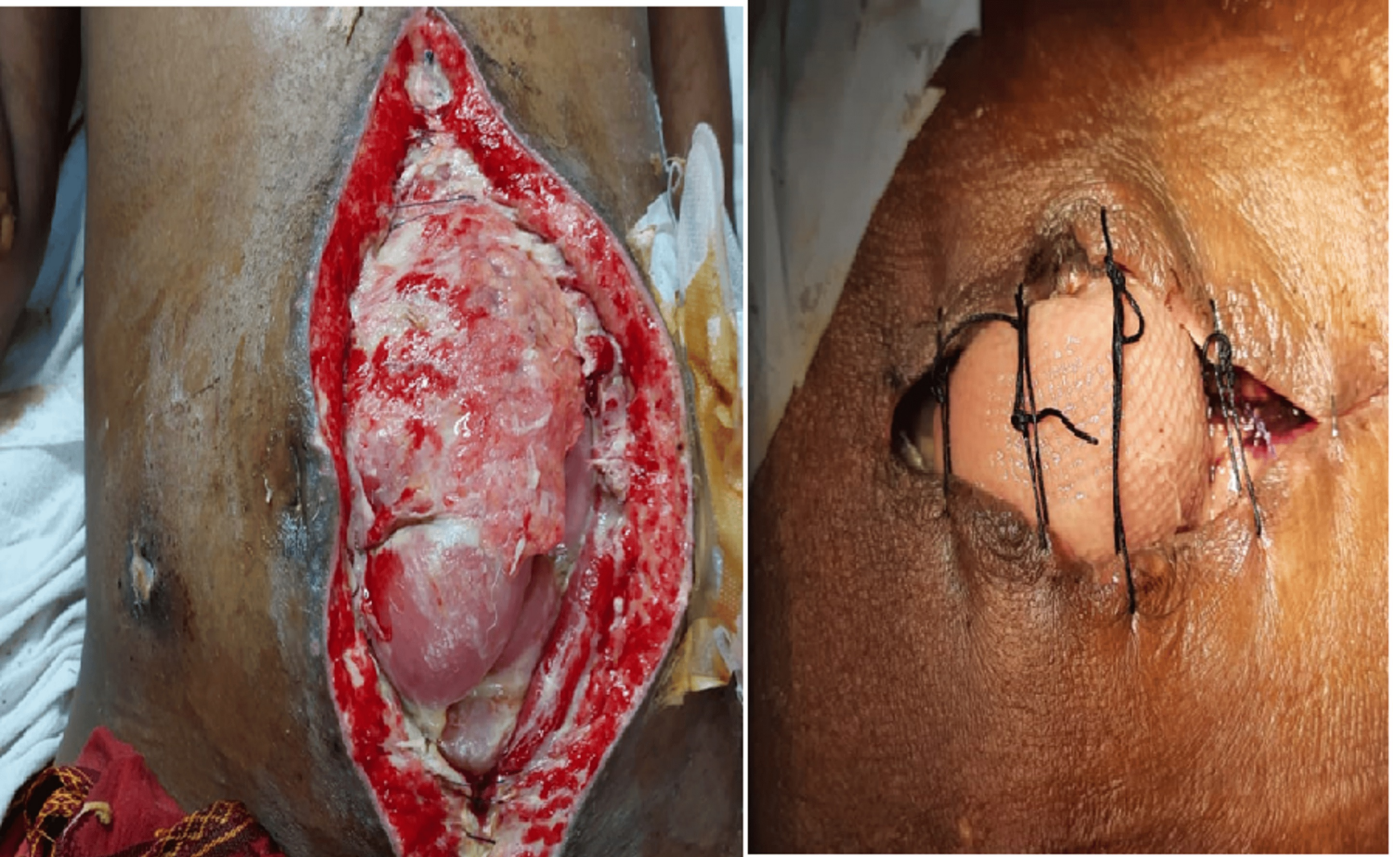
Burst abdomen

Wound infections occurred in 30 cases (42.9%), while wound dehiscence was observed in 20 cases (28.6%). Systemic inflammatory response syndrome (SIRS) was present in 18 patients (25.7%). Respiratory complications were noted in 32 patients (45.7%), and paralytic ileus was observed in 19 patients (27.1%). Residual abscesses were identified in eight patients (11.4%), and burst abdomen was documented in 12 patients (17.1%). Figure 9 illustrates a case of a burst abdomen.

A renal complication characterized by decreased urine output and elevated serum creatinine levels occurred in one patient who had previously undergone a contrast-enhanced CT scan at another facility and was referred for intestinal obstruction. During surgery, a torsed Meckel’s diverticulum, attached to the umbilicus by fibrous tissue, was identified, along with a perforation at the base of the diverticulum, necessitating resection of the unhealthy bowel with end-to-end anastomosis. Postoperatively, the patient’s urine output declined, and serum creatinine levels rose sharply, requiring hemodialysis. This complication was attributed to contrast toxicity from the prior CT scan and was deemed unrelated to the surgical procedure. Table 6 outlines the sequence of events on various postoperative days (POD).

**Table 6:** Sequence of events in the patient with contrast-induced nephropathy POD, postoperative day

POD	Urine output (mL)	Creatinine	Hemodialysis
1	200	2.4	
2	150	2.7	
3	50	4.6	Session 1
4	100	4.8	

**Figure 10:**
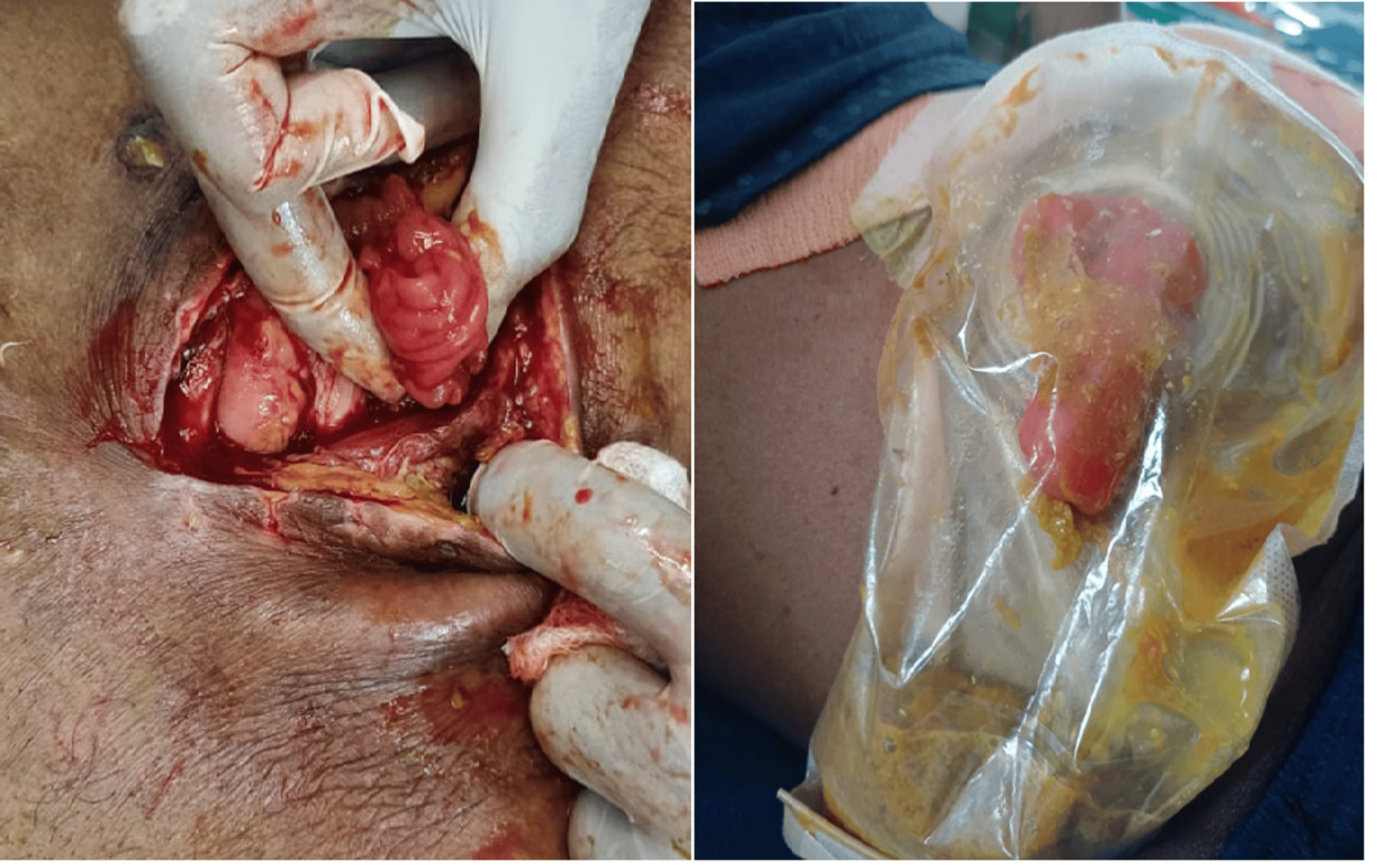
Revision surgery for stoma retraction (left) and prolapse (right)

A fecal or enterocutaneous fistula, defined as a communication between the bowel and skin, developed in three patients (4.3%). An incisional hernia occurred in one patient (1.4%). Stoma retraction and prolapse each affected one patient (1.4%). Overall, 13 deaths (18.6%) were recorded. Figure 10 illustrates stoma retraction (left) and prolapse (right).

Reperforation is defined as the presence of bowel contents in the drain after at least 48 hours post-surgery or following the introduction of oral intake. One case of reperforation occurred in the ileostomy group. The patient began oral intake on POD 2, with minimal drain output for two consecutive days, leading to drain removal on POD 5. However, on POD 7, bilious fluid was observed at the drain site, followed by a burst abdomen. Although the site of perforation was localized, no surgical repair was attempted due to bowel edema, and the patient’s condition was managed conservatively. The drain output from the perforation exceeded that from the stoma. The patient was discharged on POD 26, with follow-up still pending.

**Figure 11:**
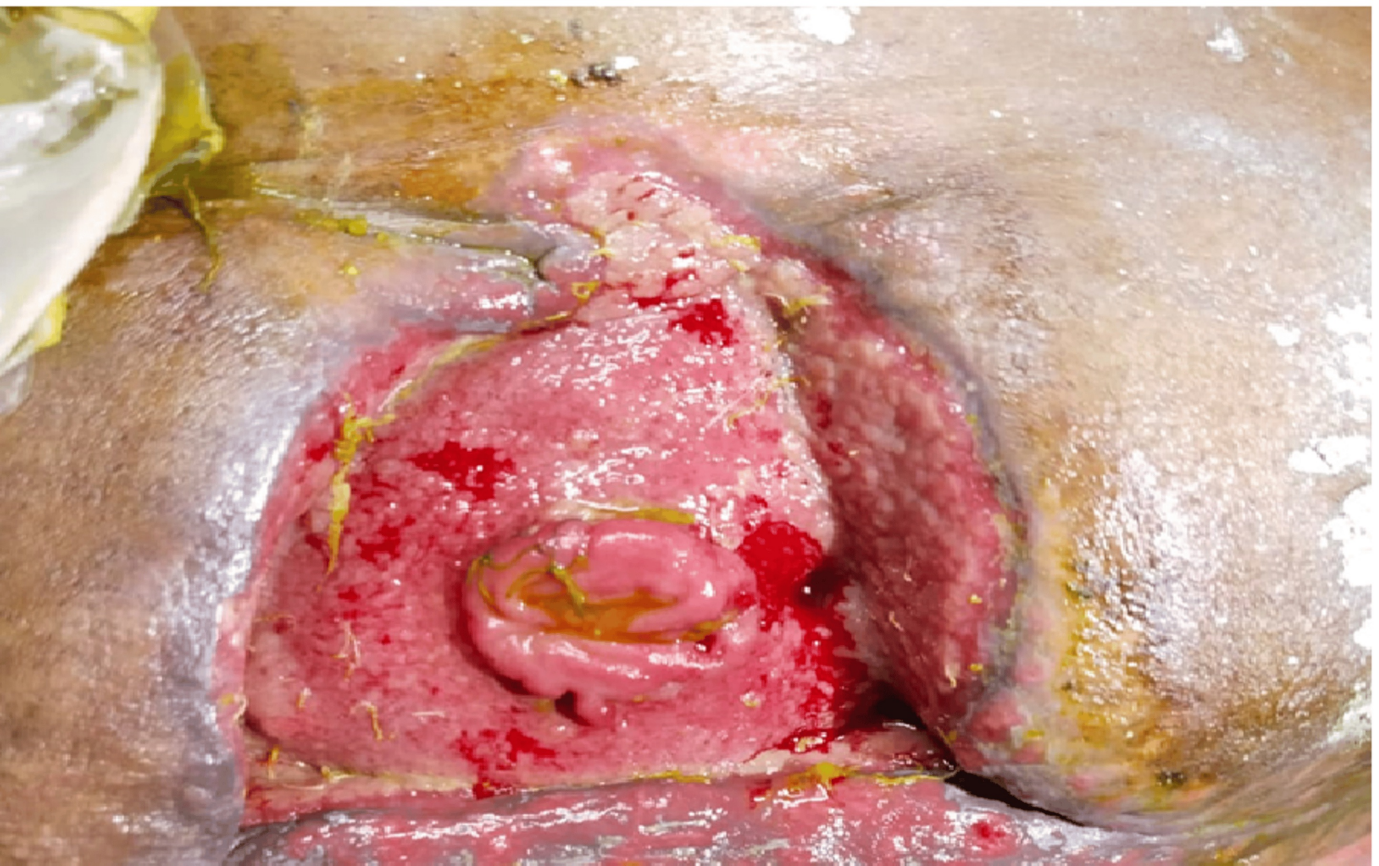
Reperforation in ileostomy cases

In a second case, reperforation occurred in a patient who had undergone closure. On POD 5, bilious fluid began leaking from the midline incision. Upon reopening the sutures, a perforation was found 2 cm proximal to the original closure site. Due to the edematous state of the bowel, the patient’s condition was managed conservatively with regular dressing, including suction dressings. The discharge gradually decreased, and the perforation eventually closed spontaneously. The patient was discharged on POD 76 after the abdominal wound was closed. In both cases, tuberculosis was the underlying diagnosis, indicating that tuberculous perforations are prone to reperforation and that minimal segmental resection offers no clear benefit. Figure 11 illustrates the reperforation.

A leak occurred in five patients (7.1%), three of whom developed fecal fistulas. Table 7 presents the relationship between the type of procedure performed, the risk of leakage, and the formation of fecal fistulas.

**Table 7:** Relationship between leak, fecal fistula, and the procedure undertaken

Column			Fecal fistula		Total
			No	Yes	
Closure	Leak	0	13	0	13
		1	2	3	5
	Total		15	3	18
Ileostomy	Leak	0	52	0	52
	Total		52	0	52

The mean duration of hospital stay was 11.49 ± 11.17 days, ranging from one to 76 days. In the closure group, the mean duration was 17.22 days, while in the ileostomy group, it was 9.50 days. Table 8 presents the percentage of various complications associated with the different procedures performed, along with the corresponding p-values.

**Table 8:** Comparison of complications in ileostomy vs. closure SIRS, systemic inflammatory response syndrome

Complication	Number	Percentage	Ileostomy group (52)	Closure group (18)	Chi-square test	p-value	95% CI
Wound infection	30	42.90%	25	5	1.497	0.2211	Lower bound: 0.31; upper bound: 0.55
Wound dehiscence	20	28.60%	17	3	0.989	0.32	Lower bound: 0.18; upper bound: 0.39
SIRS	18	25.70%	16	2	1.774	0.1829	Lower bound: 0.15; upper bound: 0.36
Respiratory complication	32	45.70%	25	7	0.16	0.6892	Lower bound: 0.34; upper bound: 0.58
Paralytic ileus	19	27.10%	15	4	0.0563	0.8125	Lower bound: 0.16; upper bound: 0.38
Residual abscess	8	11.40%	7	1	0.229	0.632	Lower bound: 0.04; upper bound: 0.19
Burst abdomen	12	17.10%	10	2	0.181	0.6708	Lower bound: 0.08; upper bound: 0.26
Renal complication	1	1.40%	0	1	0.313	0.5757	Lower bound: -0.01; upper bound: 0.04
Fecal fistula	3	4.30%	0	3	5.447	0.0196	Lower bound: -0.01; upper bound: 0.09
Stoma retraction	1	1.40%	1				Lower bound: -0.01; upper bound: 0.04
Stoma prolapse	1	1.40%	1				Lower bound: -0.01; upper bound: 0.04
Reperforation	2	2.90%	1	1	0.00055	0.9813	Lower bound: -0.01; upper bound: 0.07
Leak	5	7.10%	0	5	11.649	0.0006	Lower bound: 0.01; upper bound: 0.03
Leak leading to fecal fistula	3	4.20%	0	3	27.432	<0.0001	Lower bound: -0.08; upper bound: 1.28
Incisional hernia	1	1.40%	1	0	0.313	0.5757	Lower bound: -0.01; upper bound: 0.04
Death	13	18.60%	11	2	0.351	0.5534	Lower bound: 0.09; upper bound: 0.28

## Discussion

Out of the 70 ileal perforation cases analyzed in this study, patient ages ranged from 10 to 80 years, with a mean age of 40.83 years. Khanna and Misra reported a peak incidence in the third decade, consistent with our findings, where 19 patients (27.1%) were aged between 21 and 30 years [14]. The cohort comprised 19 females and 51 males, resulting in a male-to-female ratio of 2.68:1. This ratio closely aligns with the findings of Wani et al. (3:1) [4], Saini et al. (2.63:1) [3], and Shukla et al. (3.4:1) [18], but contrasts with the higher ratio of 6.5:1 observed in Mittal et al.’s study [1].

Among the patients with tuberculosis, the male-to-female ratio was 5:1, contrasting with the ratios of 0.9:1.9 reported by Dasgupta et al. [20] and 1:1.5 by Kumar [21]. This suggests a higher incidence of tubercular perforations in males. For typhoid cases, the male-to-female ratio was 4.33:1, similar to the ratios observed by Ugochukwu et al. (3:1) [15], Atamanalp et al. (3.6:1) [22], and Singh et al. (3:1) [23]. However, this was lower than the 11.5:1 and 8:1 ratios reported by Khanna and Misra [14] and Kapoor et al. [17], respectively.

Abdominal distension was present in all 70 patients (100%), consistent with Na’aya et al. [16] and Ansari et al. [2], who also reported a 100% incidence. However, this contrasts with the findings of Abdullah et al. [5], Ugochukwu et al. [15], Shukla et al. [18], and Mittal et al. [1], where the symptom was observed in 67%, 75.6%, 68.5%, and 83.3% of patients, respectively. A more significant difference was noted in the studies by Wani et al. [4] and Kumar [21], where only 54% and 28% of patients experienced abdominal distension.

Abdominal pain was reported by all 70 patients (100%), aligning with the findings of Na’aya et al. [16], Abdullah et al. [5], Ansari et al. [2], Wani et al. [4], Siddiqui et al. [24], Shukla et al. [18], and Mittal et al. [1], all of whom noted the symptom in 100% of their patient populations. This is comparable to the studies by Ugochukwu et al. [15] and Kumar [21], which reported abdominal pain in 90.7% and 96.67% of patients, respectively.

The mean duration of abdominal pain was 4.76 days, similar to the 5.6 days reported by Na’aya et al. and Ansari et al. [2,16]. The mean duration of abdominal distension was 3.23 days, although no studies were identified for comparison. Fever was documented in 34 patients (48.6%), consistent with findings from Ugochukwu et al. [15], Wani et al. [4], and Mittal et al. [1]. Vomiting was reported by 31 patients (44.3%), comparable to the rates observed by Na’aya et al. [16], Wani et al. [4], Shakil et al. [24], Shukla et al. [18], and Kumar [21], which reported vomiting rates of 46.5%, 42%, 44%, 46.8%, and 50%, respectively. Diarrhea was present in seven patients (10%), which is comparable to the findings of Ansari et al. [2] and Shukla et al. [18], who reported diarrhea rates of 11.36% and 11.4%, respectively. Absolute constipation was noted in 46 patients (65.7%).

A history of diabetes, hypertension, and tuberculosis was recorded in two patients (2.9%). The patients with a history of tuberculosis had completed six months of ATT. Surgical history was documented in five patients (7.1%). Of these, one patient (1.4%) had undergone hysterectomy, LSCS, and BLTL, while two patients (2.9%) had previous exploratory laparotomies, although documentation for these procedures was lacking.

In their study of 100 typhoid perforations, Khanna and Misra considered the onset of abdominal pain and distension as indicative of perforation [14]. We adopted a similar approach, except in cases with a prolonged history of abdominal distension, where the sudden onset or worsening of symptoms was used to define the time of perforation. The average time from the onset of acute abdominal symptoms to surgical intervention (lag period) was 4.204 ± 3.134 days. In patients who survived, the mean lag period was 4.017 days, compared to 5.76 days in those who did not survive, suggesting that delayed surgical intervention may contribute to mortality. Among the 25 patients who presented within 48 hours, 14 (56%) underwent ileostomy. In contrast, 38 out of 45 patients (84.4%) who presented after 48 hours required ileostomy. The mean lag period for the closure group was 2.50 days, while for the ileostomy group, it was 4.98 days, indicating that delayed presentation is an independent predictor of stoma creation. Malik et al. [13] reported mortality in three patients (12%) who presented within 48 hours, compared to 10 patients (22.2%) who presented after 48 hours. This finding is consistent with Ugochukwu et al., which demonstrated a mortality rate of 15% for early presentations and 50% for delayed presentations [15]. Wani et al. also observed increased mortality associated with delayed presentation in their study of 79 cases of non-traumatic ileal perforation, a finding echoed by Atamanalp et al. [4,22]. Similarly, Babu et al. concluded that delayed presentation adversely affects prognosis and increases the likelihood of stoma creation [6].

The mean duration of surgery was 2.707 ± 0.6941 hours. In the closure group, the mean duration was 2.889 ± 0.5830 hours, while the ileostomy group had a shorter mean duration of 2.635 ± 0.7321 hours. Thus, the ileostomy group experienced a shorter surgical duration and reduced exposure to anesthetic drugs. In the mortality group, the mean duration of surgery was three hours, compared to 2.64 hours in the non-mortality group, indicating that longer surgical durations are associated with increased mortality, consistent with observations made by Atamanalp et al. in their 2007 study on typhoid perforations [22].

Pyoperitoneum was present in 12 patients (17.1%). The mean duration of surgery for these patients was 3.3 hours, compared to 2.577 hours for those without pyoperitoneum, suggesting that its presence is associated with a longer duration of surgery. Only one patient (8.3%) with pyoperitoneum underwent primary closure; the remainder were subjected to ileostomy, indicating that pyoperitoneum may lead surgeons to defer the choice of primary closure. Mortality occurred in four patients (33.3%) with pyoperitoneum. The lag period for patients with pyoperitoneum was 5.75 days, while it was 3.22 days for those without, indicating that an increased delay in presentation is associated with pyoperitoneum and contributes to a higher likelihood of stoma creation.

Rajagopalan suggests that in cases of ileal perforation, resection of the affected segment and anastomosis should be performed for multiple perforations; however, in instances of severe contamination, ileostomy is preferred [25]. The mean number of perforations was 1.59 ± 0.970. In the closure group, the mean number of perforations was 1.33 ± 0.840, compared to 1.67 ± 1.004 in the ileostomy group. Thus, a higher number of perforations is associated with stoma creation. The maximum number of perforations observed was five. Overall, 45 patients (64.3%) had a single perforation, which is comparable to 74% reported by Abdullah et al. [5], 69% by Kim et al. [26], 67% by Kouame et al. [12], and 62% by Wani et al. [4], and significantly higher than that of Shukla et al. [18]. Additionally, 25 patients (35.7%) had multiple perforations, which is comparable to the report of Wani et al. [4] but significantly lower than that of Shukla et al. [18]. The maximum distance from the ileocecal junction where a perforation was observed was 105 cm, while the minimum distance was 10 cm. In the closure group, the minimum distance of perforation from the ileocecal junction was 15 cm, whereas in the ileostomy group, it was 10 cm.

Among patients with typhoid perforations, the mean number of perforations was 1.63 ± 1.088, with a single perforation observed in 10 of 16 patients (62.5%). This percentage is lower than that reported in other studies evaluating typhoid perforations: 90.6% by Na’aya et al. [16], 81.81% by Ansari et al. [2], 87.5% by Malik et al. [13], 81.7% by Atamanalp et al. [22], and 91% by Singh et al. [23]. Multiple perforations were identified in six patients (37.5%), higher than the 9.4% reported by Na’aya et al. [16], 18.18% by Ansari et al. [2], 12.5% by Malik et al. [13], 18.3% by Atamanalp et al. [22], and 9% by Singh et al. [23]. The maximum distance from the ileocecal junction at which perforation was observed in a patient was 105 cm.

Segment resection was performed in 35 patients (50%). Among the 12 patients with pyoperitoneum, nine (75%) underwent segment resection. In patients with typhoid, resection was performed in six patients (37.5%). Within the closure group, resection was performed in 11 patients (61.1%), while in the ileostomy group, 23 patients (44.2%) underwent resection. Closure (by primary closure or resection and anastomosis) was performed in 18 patients (25.7%), significantly lower than rates reported in other studies on ileal perforation: 94% by Abdullah et al. [5], 48.5% by Kouame et al. [12], 91.9% by Ugochukwu et al. [15], 81.9% by Ansari et al. [2], 51.8% by Malik et al. [13], and 91.8% by Babu et al. [6]. Ileostomy was performed in 52 patients (74.3%), a rate much higher than those reported by Abdullah et al. (6%) [5], Kouame et al. (51.5%) [12], Ugochukwu et al. (51.5%) [15], Ansari et al. (18.1%) [2], and Babu et al. (8.2%) [6]. Among the 52 patients who underwent ileostomy, eight (15.4%) had a double-barrel ileostomy, 15 (28.8%) had an end ileostomy, and 29 (55.8%) had a loop ileostomy.

HPE revealed the cause of perforation in 34 patients (48.6%). Findings included Crohn’s disease in one patient (1.4%), lymphocytic infiltration in two patients (2.9%), reactive lymph nodes in 12 patients (17.1%), tuberculosis in 11 patients (15.7%), and findings suggestive of typhoid in two patients (2.9%). The male-to-female ratio among patients with diverticula was 1:1, contrasting with Thilakawardana et al., which indicated that males were affected twice as frequently [27].

The Widal test was positive in 16 patients (22.9%), with a dilution titer > 1:320 considered positive. Kim et al. reported a positivity rate of 75% with significant titers defined as above a 1:80 dilution [26], while Khanna and Misra found positive titers in 58% of patients with a significant titer at 1:80 dilution [14]. Our findings align with those of Kapoor et al., who observed a positivity rate of 35% with significant titers of 1:320 dilution [17]. Thus, higher dilutions decrease the sensitivity of the Widal test but enhance its specificity, indicating that our study may have underestimated the actual number of typhoid perforations.

Nonspecific causes accounted for ileal perforations in 28 patients (40%), comparable to 26% reported by Wani et al. [4], 20.74% by Abdullah et al. [5], and 24.5% by Babu et al. [6], but significantly lower than the 70% reported by Mittal et al. [1]. Typhoid was responsible for ileal perforations in 16 patients (22.9%) in our study, comparable to Mittal et al. [1], but considerably lower than 62% reported by Wani et al. [4], 71.95% by Abdullah et al. [5], 50% by Saini et al. [3], and 64.5% by Babu et al. [6]. This discrepancy may largely stem from variations in the dilution of the Widal titer considered significant across studies. Tuberculosis caused perforations in 12 patients (17.1%), which is comparable to 10% reported by Mittal et al. [1], but higher than 3.1% by Babu et al. [6] and 3% by Wani et al. [4], yet lower than 35% reported by Saini et al. [3]. Trauma accounted for six perforations (8.6%) in our study, similar to 5% reported by Saini et al. [3] and close to 10% reported by Mittal et al. [1]. Other causes included diverticulosis in two patients (2.9%), band adhesion in two patients (2.9%), Crohn’s disease in one patient (1.4%), Meckel’s diverticulum in one patient (1.4%), non-Hodgkin’s lymphoma in one patient (1.4%), and tubulovillous adenoma in one patient (1.4%).

The most common complication observed was wound infection, which occurred in 30 cases (42.9%). This rate is comparable to the 48.8% reported by Na’aya et al. [16], 35.4% by Babu et al. [6], 36.67% by Mittal et al. [1], 29.3% by Atamanalp et al. [22], and 28% by Singh et al. [23]. However, it is significantly lower than the rates of 74%, 71.4%, 63.6%, and 68.18% reported by Khanna and Misra, Kouame et al., Ugochukwu et al., and Ansari et al., respectively [2,12,14,15]. Additionally, it is higher than the 12.1% reported by Abdullah et al. [5], 10.7% by Malik et al. [13], and 17.7% by Shukla et al. [18].

Wound dehiscence occurred in 20 cases (28.6%), which is comparable to the 27.2% reported by Ansari et al. [2]. This rate is lower than the 42% observed by Khanna and Misra [14] but significantly higher than the 9.3% reported by Na’aya et al. [16], 3.6% by Abdullah et al. [5], 15% by Ugochukwu et al. [15], 8.9% by Malik et al. [13], 11.4% by Shukla et al. [18], 18.2% by Atamanalp et al. [22], and 15% by Singh et al. [23]. SIRS was present in 18 cases (25.7%).

Respiratory complications occurred in 32 cases (45.7%), significantly higher than the rates reported in other studies: 18% by Khanna and Misra [14], 4.5% by Ugochukwu et al. [15], 8.03% by Malik et al. [13], 16.6% by Babu et al. [6], and 6.25% by Shukla et al. [18]. Paralytic ileus was observed in 19 cases (27.1%), similar to the 22% reported by Kapoor et al. [17] but higher than the 15% reported by Singh et al. [23]. Burst abdomen occurred in 12 cases (17.1%), exceeding the rates reported by other studies: 10% by Khanna and Misra [14], 9% by Kapoor et al. [17], 4.2% by Babu et al. [6], and 7.2% by Shukla et al. [18].

Renal complications were noted in one case (1.4%), significantly lower than the 15.9% reported by Kouame et al. [12] and 11.1% by Chirantan et al. [7]. Stoma retraction was observed in one case (1.9% of all ileostomy cases), comparable to the 0.89% reported by Malik et al. [13] but much lower than the 5% reported by Ansari et al. [2], 12.5% by Saini et al. [3], and 6.66% by Mittal et al. [1]. Stoma prolapse occurred in one case (1.9% of ileostomy cases), which is comparable to the 1.66% reported by Mittal et al. [1] and 1.7% by Malik et al. [13], but significantly lower than the 12.1% reported by Kouame et al. [12], 7% by Ansari et al. [2], and 5% by Saini et al. [3].

Reperforation was found in two cases (2.9%), significantly lower than the 8.5% reported by Atamanalp et al. [22]. Leakage was observed in five cases (7.1%). Fecal fistula occurred in 4.3% of cases, similar to the 4% reported by Khanna and Misra [14], 7% by Abdullah et al. [5], 3% by Ugochukwu et al. [15], 6.25% by Shukla et al. [18], and 4.6% by Babu et al. [6]. Our results were significantly lower than the 20% reported by Kapoor et al. [17], 34.9% by Kouame et al. [12], 13.36% by Ansari et al. [2], 11.1% by Chirantan et al. [7], and 9.8% by Malik et al. [13]. Incisional hernia was observed in one case (1.4%), significantly lower than the 21.5% reported by Kouame et al. [12], 36.36% by Ansari et al. [2], and 7.2% by Babu et al. [6].

The mean hospital stay was 11.49 ± 11.177 days. In the closure group, the mean stay was 17.22 ± 19.219 days, while in the ileostomy group, it was 9.50 ± 5.500 days. Mittal et al. included the duration of ileostomy reversal in their reported total hospital stay, resulting in a mean of 14.3 days for primary closure and 21.53 days for the stoma group [1].

Death occurred in 13 cases (18.6%), comparable to the 18% reported by Ugochukwu et al. [15], 14% by Na’aya et al. [16], 13.36% by Ansari et al. [2], 11.1% by Chirantan et al. [7], and 11% reported by both Atamanalp et al. [22] and Singh et al. [23]. However, it is significantly higher than the 7.8% reported by Babu et al. [6], 7.14% by Malik et al. [13], and 8% by Abdullah et al. [5], but much lower than the 47% reported by Khanna and Misra [14]. Among the 13 deceased patients, five were female and eight were male, indicating a higher mortality rate in males. This contrasts with the findings of Wani et al., who observed higher mortality in females [4]. The mean age of patients in the mortality group was 43.92 years, compared to 61.65 years in the non-mortality group. A significant p-value was noted in cases involving leaks, fecal fistulas, and leaks leading to fecal fistula.

The study’s limitations include a small sample size and its design as a single-center study. A considerable number of cases could not be included due to the lack of Widal and HPE reports. The study aimed to assess the etiology, management, and complications of ileal perforations in Jharkhand; thus, the results cannot be generalized to a broader population. While follow-up was conducted for all cases of ileostomy, as these patients returned for reversal, significant loss to follow-up occurred in the closure group. Furthermore, we did not include the duration of hospital stay for the ileostomy group when the patients returned for stoma reversal.

## Conclusions

No significant difference was observed in the complication rates across the various surgical procedures performed. However, delayed presentation was associated with higher complication rates and increased mortality. Therefore, cases presenting after 48 hours should be considered for ileostomy. In patients with tuberculosis, reperforation is common; hence, ileostomy should be considered the procedure of choice, and resection of the abnormal-looking segment should also be performed, as exteriorization of the perforation alone is insufficient.

Pyoperitoneum was associated with higher mortality compared to the non-pyoperitoneum group, indicating that stoma placement (which is associated with shorter anesthetic drug exposure) should be considered in these cases. Leaks that exhibit a decreasing output trend can be managed conservatively, while other leaks necessitate reexploration, with the anastomosis being taken down in such situations. In these instances, exteriorizing the bowel is recommended, and immediate intervention is essential. Given that no significant difference was observed between the closure and stoma groups, primary closure should be undertaken if the patient’s general condition permits and in the absence of pyoperitoneum.
